# Death of Professor Graham, of Edinburg

**Published:** 1845-10

**Authors:** 


OBITUARY.

*The late Professor Graham of Edinburgh.*—It is with sincere sorrow that we announce the death of this distinguished individual. The mournful event took place at Coldoch, in Perthshire, on the 7th of August, after a painful and protracted illness, which he bore with calmness and Christian fortitude.

Robert Graham, M.D., F.R.S.E., Professor of Botany and Medicine in the University of Edinburg, the third son of Dr. Robert Graham, afterwards Moir of Leckie, was born at Sterling, on 7th December, 1786.

In the first part of his career, he practised in Glasgow, where he was highly respected, and very popular. In 1818, he was appointed Professor of Botany in the University of that city. Previous to that time, there was no separate chair of Botany in Glasgow. The Professor of Anatomy, by his commission, was also Professor of Botany: he was bound to lecture on Anatomy during the winter, and on Botany during the summer session. Dr. Jeffray, the present Professor of Anatomy, lectured occasionally on Botany; but subsequently, a separate lectureship was established. Dr. Thomas Brown of Langfine held this office for some time. Before retiring, he asked Dr. Graham to lecture for him, which Dr. Graham declined to do, urging as an apology, the inadequacy of his Botanical knowledge; but ultimately, he was prevailed on to read Dr. Brown's lectures. On the resignation of Dr. Brown, the Crown instituted a distinct chair of Botany, and conferred it upon Dr. Graham, who was in the habit of referring to this appointment as an unexpected event, on which his future success in life depended. He held this office till his translation to the chair of Botany in the University of Edinburgh, in 1821. From this time, Dr. Graham devoted himself assiduously and successfully to Botanical pursuits. To his exertions, Edinburgh is in no small degree indebted, for the excellent Botanical Garden which she now possesses. By his enthusiasm and energy, as well as by his affable and pleasing manners, he did much to promote a taste for his favourite science, among the pupils of his class.—*London and Edinburgh Monthly Journal.*

